# Whole-plant trait networks reveal elevational optimization of resource strategies: integration drives distribution in woody saplings

**DOI:** 10.3389/fpls.2025.1463237

**Published:** 2025-09-11

**Authors:** Zuhua Wang, Haibo Li, Chuandong Yang, Jugang Wang, Fen Chen, Min Liu

**Affiliations:** ^1^ College of A&F Engineering and Planning, Tongren University, Tongren, Guizhou, China; ^2^ Guizhou Provincial Key Laboratory for Biodiversity Conservation and Utilization in the Fanjing Mountain Region, Tongren University, Tongren, Guizhou, China; ^3^ National Nature Reserve Administration of Fanjing Mountain, Tongren, Guizhou, China

**Keywords:** plant trait networks, elevation gradient, climate regulating, trait integration, species distribution

## Abstract

**Introduction:**

Plant trait networks (PTNs) reveal integrated adaptation strategies, but how elevational stress gradients reshape PTN architecture and influence species distribution remains unclear.

**Methods:**

We analyzed 14 leaf, stem, and root traits across 37 woody sapling species along a 600 – 2200 m elevational gradient on Mt. Fanjingshan, China. We linked PTN metrics (connectivity, modularity, hub traits) to environmental drivers and species distributions.

**Results:**

PTN integration increased with elevation, evidenced by declining average path length (R² = 0.93, *P* = 0.008) and graph diameter (R² = 0.92, *P* = 0.011), indicating intensified trait coordination at higher elevations. Modularity peaked at mid-elevations (unimodal pattern: R² = 0.97, *P* = 0.017), reflecting heightened trade-offs between stress tolerance and resource acquisition. Crucially, litter depth and soil phosphorus—not climate—were the primary drivers of PTN structure, jointly explaining 84.2% of variation (*P* = 0.011) and promoting integration via root-hub traits (specific root length, specific root area). Species distribution was strongly correlated with PTN efficiency (84.8% explained variance, *P* = 0.038), driven by reduced graph diameter, greater litter depth, and lower temperature.

**Discussion:**

These findings indicate that elevational stress selects for highly integrated PTNs optimized by belowground trait hubs and microhabitat buffering, highlighting litter-soil interactions as critical mediators of species distributions under climatic constraints.

## 1 Introduction

Plant trait networks (PTNs) provide a transformative framework for decoding plant adaptive complexity by quantifying interdependencies among functional traits. In PTNs, nodes represent traits and edges reflect trait correlations, revealing how plants optimize resource acquisition under environmental constraints (He et al., 2020). Crucially, key PTN properties encode critical ecological trade-offs: high network connectivity (characterized by low average path length and diameter) enhances resource-use efficiency (Rao et al., 2023); high modularity (reflected in a high clustering coefficient) facilitates specialized stress responses (Li et al., 2022); and hub traits (with high betweenness centrality) dictate strategic resource allocation priorities (Rao et al., 2023). Saplings, with heightened sensitivity to climate shifts and vital roles in forest regeneration and biodiversity maintenance (Kirk et al., 2021; Wang et al., 2022a; Yang et al., 2023), therefore offer a critical lens for studying these adaptive mechanisms. While functional traits are established indicators of plant strategies (Green et al., 2022; Wang et al., 2022b; Wang and Ali, 2021), research has predominantly focused on adults, leaving a significant gap in understanding sapling adaptation through the lens of PTNs.

Elevational gradients function as natural laboratories where temperature, resource availability, and biotic interactions vary predictably together (Weemstra et al., 2022), enabling tests of how PTNs respond to environmental stressors. At low elevations, intense light competition drives carbon allocation toward photosynthetic structures, potentially constraining overall trait network integration (de la Riva et al., 2021; Sierra Cornejo et al., 2020; Flores-Moreno et al., 2019). Conversely, high-elevation saplings face nutrient limitation and cold stress while benefiting from reduced canopy cover and litter-mediated soil insulation (Ameztegui et al., 2021; Wang et al., 2022b; Kaspari and Yanoviak, 2008). These conditions are predicted to select for highly connected PTNs that maximize growth efficiency during brief favorable growing periods (Weemstra et al., 2020; Wang et al., 2023). Concomitant shifts in hub traits—those possessing high network centrality—are expected. Specifically, hub traits likely shift from leaf traits (e.g., specific leaf area), which prioritize light capture at low elevations, to root traits (e.g., specific root length, specific root area), which optimize nutrient foraging at high elevations (Wang et al., 2025). Critical knowledge gaps persist regarding how PTN architecture in saplings responds to elevational stressors and whether PTN properties can predict species distribution patterns. Understanding this nexus is essential for biodiversity forecasting. However, current trait-based distribution models yield inconsistent results (Bueno et al., 2023; van der Plas et al., 2023), likely due to their oversimplification of the trait synergies inherent in PTNs.

To address these gaps, we leverage Mt. Fanjingshan’s 2000-m elevational gradient—a UNESCO World Heritage Site with pronounced climatic and edaphic zonation supporting exceptional biodiversity (Wang et al., 2022b, 2023). We integrate meteorological data, soil analyses, and functional traits of 164 woody saplings (37 species) across six elevations to test five hypotheses: (1) Trait network integration increases with elevation, reflected in shorter average path lengths and reduced graph diameter due to stronger environmental filtering; (2) Modularity follows a nonlinear (hump-shaped) relationship with elevation, peaking where trade-offs between stress tolerance and resource acquisition are most acute; (3) hub traits transition from leaf traits (low elevation) to absorptive root traits (high elevation) as resource constraints shift; (4) Species distribution is associated with efficient PTNs (shorter path lengths, smaller diameters), indicating optimized trait combinations for habitat occupancy. By testing these hypotheses, we advance understanding of how multidimensional drivers shape trait coordination and community assembly along environmental gradients.

## 2 Materials and methods

### 2.1 Study site and design

The study was conducted at Mt. Fanjingshan (27.78 – 28.02°N, 108.60 – 108.81°E) in northeastern Guizhou Province, Southwest China, characterized by a humid subtropical monsoon climate with mean annual temperatures of 5.0 – 17.0°C and annual precipitation of 1100 – 3000 mm. Encompassing an elevational gradient exceeding 2000 m, the mountain exhibits a well-defined vertical vegetation zonation comprising five distinct belts: evergreen broad-leaved forest, evergreen-deciduous broad-leaved mixed forest, deciduous broad-leaved forest, subalpine coniferous forest, and alpine shrub meadow (Wang et al., 2022b). Soils are predominantly mountainous yellow soil or yellow-brown soil (classified as Dystric Cambisols under the FAO system), with silty loam texture. Six sampling sites without disturbance were established on relatively uniform slopes devoid of major ridges or valleys, positioned at 600 m (evergreen broad-leaved forest dominated by *Litsea elongata* and *Litsea pedunculata*), 1100 m (evergreen broad-leaved forest with *Symplocos sumuntia* and *Cyclobalanopsis sessilifolia* as codominants), 1480 m (evergreen-deciduous broad-leaved mixed forest dominated by *Lindera fragrans*), 1700 m (evergreen-deciduous broad-leaved mixed forest dominated by *Camellia cuspidata*), 2000 m (deciduous broad-leaved forest with codominant *Camellia cuspidata* and *Camellia japonica*), and 2200 m (evergreen-deciduous broad-leaved mixed forest where *Rhododendron auriculatum* predominates).

### 2.2 Field methods and calculations

At each site, a transect parallel to the elevation contour contained 10 sampling points spaced 15 m apart (Wason and Dovciak, 2017) (**Supplementary Figure S3**). Using the point-centred-quarter method (Mitchell, 2007) to investigate all woody species trees >2 cm in diameter at breast height (DBH) at each sampling point. A cross-shaped frame oriented with a transect expansion direction as the x-axis defined the local coordinate system, dividing the area into four quadrants. Within each quadrant, the nearest woody plant (>2 cm DBH) was selected as the target species. And then, saplings (2 cm ≤ DBH < 10.2 cm) followed the classification of Wason and Dovciak (2017). In this study, we sampled 164 sapling individuals belonging to 37 species, with 3 – 5 individuals per species (**Supplementary Table S1**). For every selected plant, we recorded species identity and measured: (i) distance from base to sampling point, (ii) DBH, and (iii) height. Following Mitchell (2007), we calculated tree density, basal area, relative frequency, and importance value for each species, where importance value = Relative density + Relative cover + Relative frequency. Specifically:

Relative density (Species k) = [Occurrences of species k/(4 × n)] × 100.

(n = number of sampling points; 4n = total possible occurrences).

Relative cover (Species k) = [Total basal area of species k/Total basal area of all species] × 100.

Relative frequency (Species k) = [Absolute frequency of species k/Total absolute frequency of all species] × 100.

### 2.3 Sampling and trait measurement

Sampling was conducted between July and August 2022, and one terminal, fully expanded, sun-exposed branch was selected from the current growing season from each dominant species. The branches were cut using a 5.6 m telescoping pole (ARS Corp., Senboku, Japan), labeled, and then placed in a cooler for transport to the laboratory. At the same time, roots were sampled from the uppermost 20 cm of soil by tracing the coarse roots of a target tree from the trunk until strands of fine roots (diameter < 2 mm) were reached. Then, the roots from each tree with intact root networks (i.e., including the first five orders) were collected. Each root sample was placed into ice bags and then stored at -20 °C for later dissection and analyses of morphological and chemical traits. Simultaneously, we measured the tree height (TH, abbreviations in **Table 1**) of each target tree using a graduated pole (maximum graduation: 15 m).

**Table 1:** Variation in fourteen leaf and fine-root traits measured in 37 tree species.

Traits	Abbreviations	Unit	Mean	SD	Min	Max	Blomberg’s *K*	*P* value
Tree height	TH	m	5.00	1.81	1.72	10.07	0.16	0.09
Root dry matter content	RDMC	g/g	0.25	0.06	0.12	0.43	0.12	0.55
Specific root length	SRL	m/g	44.93	28.8	12.73	147.35	0.11	0.58
Root tissue density	RTD	m^3^/g	0.94	0.51	0.26	2.95	0.08	0.82
Root diameter	Rdia	mm	0.23	0.06	0.07	0.47	0.12	0.42
Specific root area	SRA	cm^2^/g	290.67	180.61	131.12	1050.54	0.09	0.69
Root N	RN	%	2.05	0.43	1.05	3.13	0.17	0.20
Root C	RC	%	48.09	2.23	43.06	53.63	0.09	0.72
Root P	RP	g/kg	0.69	0.29	0.33	1.95	0.43	0.06
Specific leaf area	SLA	cm^2^/g	168.63	79.96	70.07	553.04	0.26	0.05
Leaf dry matter content	LDMC	g/g	22.36	7.71	3	37	0.15	0.24
Leaf N	LN	g/g	2.13	0.6	1.32	4.05	0.18	0.14
Leaf C	LC	%	47.69	3.33	39.02	52.07	0.12	0.08
Leaf P	LP	g/kg	0.83	0.45	0.33	2.67	0.08	0.21

SD, is standard deviation; Max, is maximum; and Min, is minimum.

Phylogenetic signal and significance were tested using a two-sided Blomberg’s K test.

In the laboratory, the branches were placed in water to minimize leaf dehydration, and 20 leaves were selected from each branch to measure for fresh weight. These leaves were separately laid flat and imaged together with a reference square (4 cm^2^) using an EPSON perfection V700 scanner (EPSON America Inc.), and the total projected leaf area was calculated using the image-processing software ImageJ (Systat Software Inc., Richmond, CA). Then, fine roots were stored in deionized water. The larger intact roots were carefully removed from the soil with a pair of forceps, and the remaining soil on the roots was brushed away and dissected as described by (Pregitzer et al., 2002). The most distal root tips with no branches were defined as the first order, and the roots in which two first-order roots intersected comprised the second order. The remaining branch orders were determined similarly. We classified the absorptive roots as first- and second-order roots (McCormack et al., 2015). Then, 60 absorptive roots per species were measured for fresh weight and subsequently scanned using an EPSON perfection V700 scanner (EPSON America Inc.), and the captured images were analyzed to determine the diameter (Rdia), length, and surface area using WinRHIZO Version 2005c (Regent Instrument Inc., Nepean, ON, Canada).

After scanning, the leaves and roots were dried in a forced-air oven at 70 °C for 48 hours, followed by weighing, homogenization using a coffee mill, grinding, and sieving through a 0.15 mm mesh. Leaf nitrogen concentration (LN), leaf carbon concentration (LC), root carbon concentration (RC), and root nitrogen concentration (RN) were determined using an elemental analyzer (Vario EL III, Germany). The molybdate/ascorbic acid method was applied to measure the total phosphorus concentrations in the leaves (LP) and absorptive roots (RP) after H_2_SO_4_–H_2_O_2_ digestion (Jones, 2001). Leaf dry matter content (LDMC) and root dry matter content (RDMC) were calculated as dry mass divided by fresh mass (g/g). Specific leaf area (SLA) was calculated as the total leaf surface per unit dry mass (cm^2^/g). Specific root length (SRL) was estimated as root length divided by root biomass (m/g). Root tissue density (RTD) was estimated as the ratio of root biomass to root volume, assuming a cylindrical shape (g/m^3^), and specific root area (SRA) was calculated as root surface area divided by root biomass (cm^2^/g).

### 2.4 Soil properties and climate properties

For every target tree across all sampling sites, a 1 × 1 m subplot was established centered on the tree. Within each subplot, five random points were designated for soil property measurements. Litter depth (LD, cm) and soil depth (SD, cm) were measured using a ruler, with SD additionally assessed using a steel stick. Soil pH (SpH) was determined using a pH meter. Soil temperature (ST) and soil humidity (SH) were recorded at three random points per subplot with a portable soil sensor (TZS-ECW-G). Fresh soil samples collected from each sampling point were immediately placed in sealed bags, refrigerated, and transported to the laboratory within 24 hours. Total soil nitrogen (N) was analyzed using an elemental analyzer (Vario EL III, Germany), while total soil phosphorus (P) concentrations were measured via perchloric acid digestion followed by molybdate colorimetry. Since 2018, a meteorological observation station has been operational at each of the six elevations. Consequently, the mean daily values for mean daily air temperature (AT), air humility (AH), and air pressure (AP) for each elevation were calculated from the Mt. Fanjingshan Meteorological Station records.

### 2.5 Data analysis

For the plant trait network analysis encompassing 14 traits (**Table 1**), traits served as nodes and significant pairwise correlations formed edges. Using the V. PhyloMaker package (Jin and Qian, 2022), we first assessed phylogenetic signals but detected none (**Table 1**). We then calculated Pearson correlations among traits within each elevational band. To minimize spurious relationships, only correlations significant at *P* < 0.05 were retained; absolute correlation coefficients (|r|) defined edge strength. PTNs for each elevation were constructed and visualized using the igraph package (Csardi and Nepusz, 2006). For network connectivity metrics, we calculated graph diameter and average path length using igraph’s diameter and average.path.length functions, respectively. For community structure, we identified modules and calculated modularity using the cluster_spinglass algorithm. The average clustering coefficient was computed with igraph’s transitivity function. Hub traits per elevation were identified based on high node betweenness centrality, indicating pivotal network positions.

Effects of environmental factors on PTN metrics (hub traits and structure) was used a Redundancy Analysis (RDA; vegan package, Oksanen et al., 2022). Initial explanatory variables comprised climate (AT, AP, AH) and soil properties (SN, SP, SD, LD, ST, SH). To avoid collinearity, pairwise Pearson correlations among predictors were calculated. Variables exhibiting correlation coefficients > 0.70 were excluded **Supplementary Figure S2**), resulting in the final retained predictors: AT, AH, SP, and LD. The relative contributions of these retained environmental controls were further partitioned using the varpart() function (vegan). Separate RDA models examined the influence of PTN metrics (graph diameter), climate (AT), and soil properties (LD) on species distribution (represented by tree density and species importance value), identifying graph diameter, AT, and LD as the key retained predictors. Additionally, polynomial regression models tested variations in PTN metrics, climate variables, and soil properties across the elevation gradient. Model assumptions were verified by checking for outliers and assessing the normality, heterogeneity, and homogeneity of residuals (Zuur et al., 2009); variables were log-transformed where necessary to meet these assumptions.

All calculations were performed using R software (v 4.2.1, R Core Team 2022), and significant effects were detected at the level of *P*<0.05.

## 3 Results

### 3.1 Variations in soil properties and climate with elevation

Soil nitrogen (R² = 0.15, *P* = 0.004), soil phosphorus (R² = 0.08, *P* = 0.031), soil depth (R² = 0.23, *P* = 0.002), and soil humidity (R² = 0.29, *P* < 0.001), as well as litter depth (R² = 0.27, *P* < 0.001), significantly increased with elevation **Supplementary Figures S1a–c, e, h)**. In contrast, soil pH (R² = 0.49, *P* < 0.001), soil temperature (R² = 0.86, *P* < 0.001), air pressure (R² = 0.98, *P* < 0.001), and air temperature (R² = 0.98, *P* < 0.001) decreased significantly with increasing elevation **Supplementary Figures S1d, f, k, i**). Air humidity exhibited a significant U-shaped relationship with elevation (R² = 0.75, *P* < 0.001, **Supplementary Figure S1j**). Both species importance value **Supplementary Figure S1g**) and tree density **Supplementary Figure S1l**) exhibited significant positive correlations with elevation (importance value: *R*² = 0.20, *P* = 0.002; tree density: *R*² = 0.44, *P* < 0.001).

### 3.2 Variations in trait network metrics with elevation

**Figure 1:**
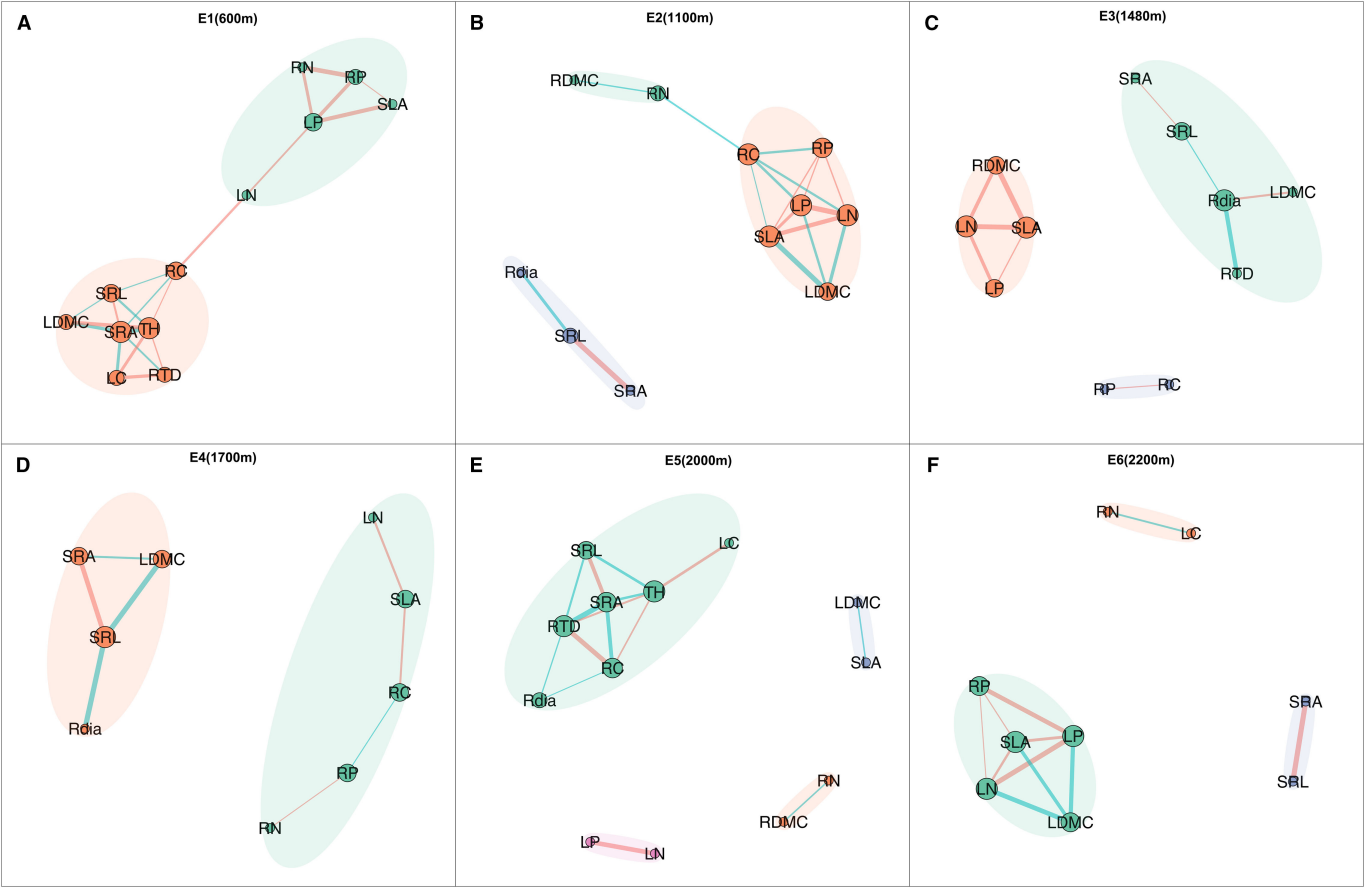
Plant trait networks (PTNs) across species at six elevations: **(A)** 600 m, **(B)** 1100 m, **(C)** 1480 m, **(D)** 1700 m, **(E)** 2000 m, and **(F)** 2200 m. Within each panel, modules within the PTN are color-coded. Edges represent significant trait correlations, with red and green lines indicating positive and negative correlations, respectively. Node size corresponds to node degree. Traits showing no significant correlations with others are omitted from the visualization. Trait abbreviations are defined in **Table 1**.

**Figure 2:**
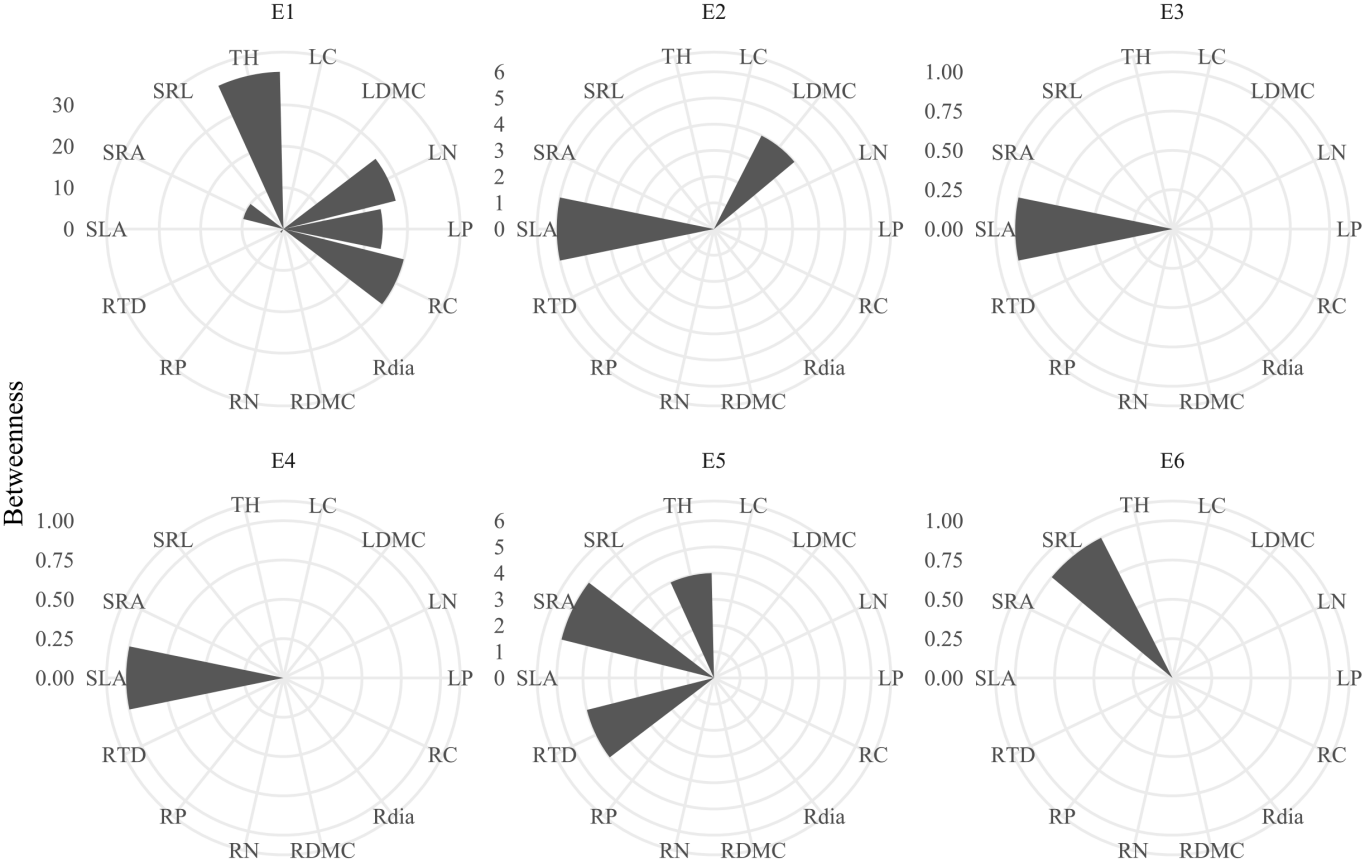
Betweenness centrality in plant trait networks across the elevational gradient. Trait abbreviations are provided in **Table 1**. Labels E1–E6 correspond to sampling elevations: 600 m, 1100 m, 1480 m, 1700 m, 2000 m, and 2200 m.

**Figure 3:**
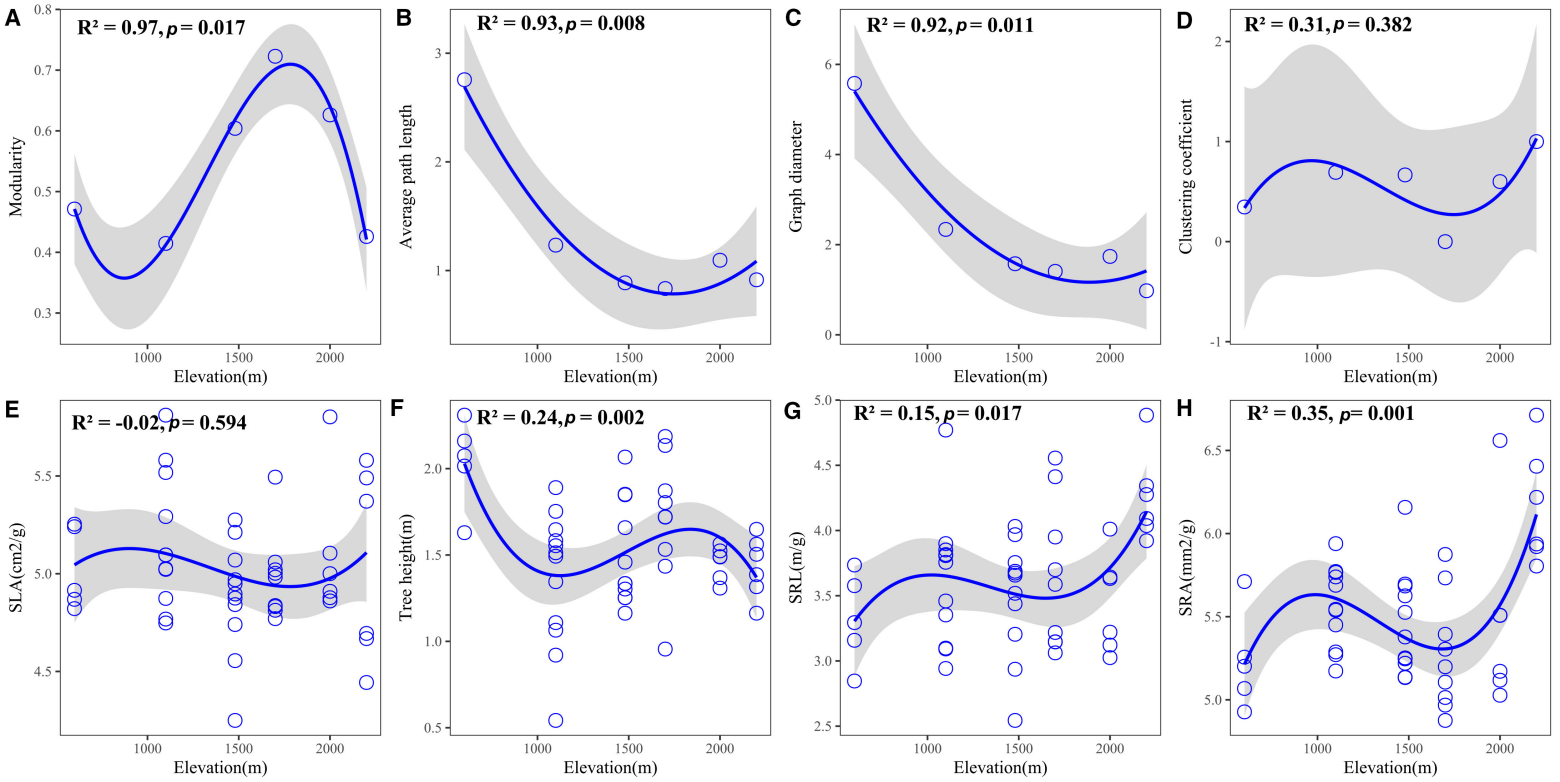
Variation in **(A–D)** plant trait network metrics and **(E–H)** hub traits across the elevational gradient. Shaded grey areas indicate 95% confidence intervals.

Plant trait networks exhibited significant elevational variations in connectivity and complexity, reflecting whole-plant coordination strategies between above- and belowground organs for environmental adaptation **Figure 1**). Hub traits varied across elevations: tree height at 600 m (E1), SLA at 1100 m (E2) and 1480m (E3), SLA at 1700 m (E4), SRA at 2000 m (E5), and SRL at 2200 m (E6) **Figure 2**). Elevational increases were associated with significant reductions in average path length (R² = 0.93, *P* = 0.008) and graph diameter (R² = 0.92, *P* = 0.011) **Figures 3B, C**). Network modularity exhibited an inverted U-shaped relationship with elevation (R² = 0.97, *P* = 0.017; **Figure 3A**), while the average clustering coefficient showed no elevational trend **Figure 3D**, P > 0.05). Among hub traits: specific root length (R² = 0.10, *P* = 0.013) and specific root area (R² = 0.24, *P* = 0.001) increased nonlinearly with elevation **Figures 3G, H**), tree height decreased linearly (R² = 0.10, *P* = 0.016; **Figure 3F**). In contrast, specific leaf area demonstrated no significant elevational pattern **Figure 3E**, *P* > 0.05).

### 3.3 Controls on the trait network structure

**Figure 4:**
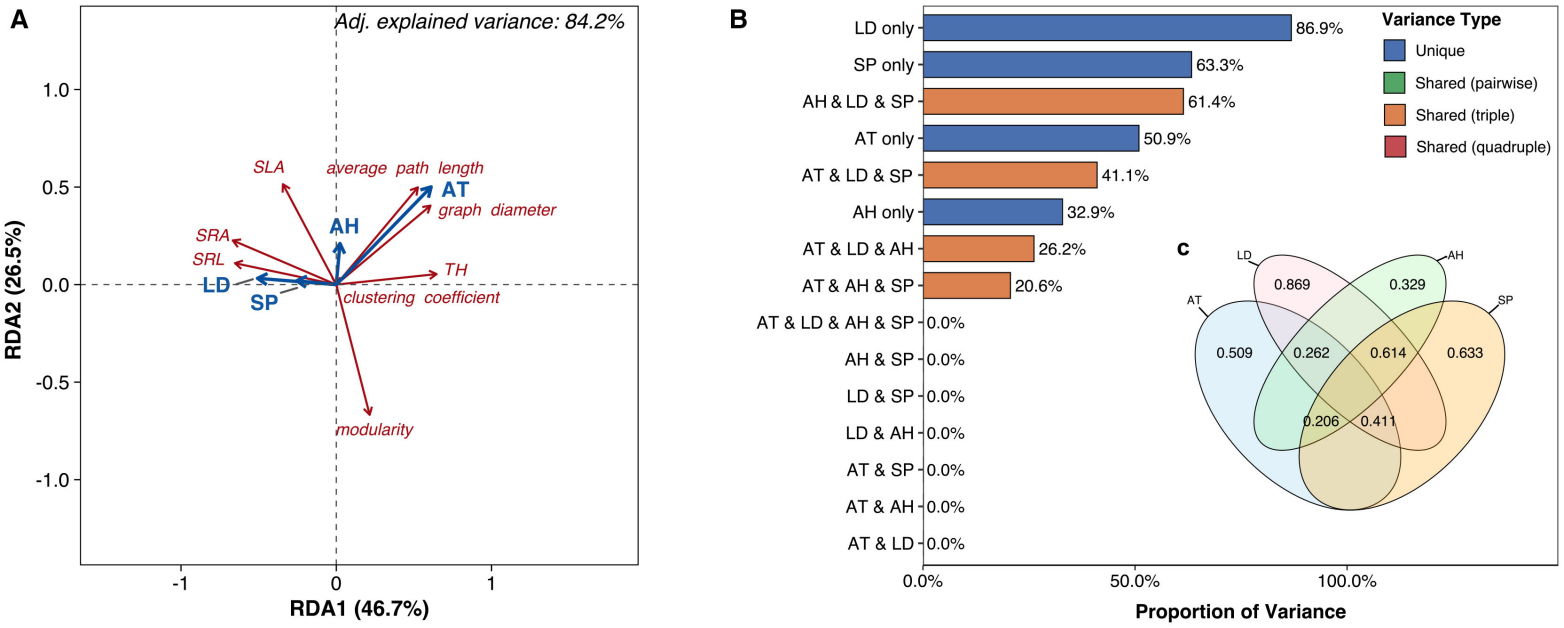
**(A)** Biplot from redundancy analysis (RDA) examining relationships between plant trait network metrics (red text labels) and environmental predictors (blue vectors). **(B)** Bar diagram and **(C)** variance partitioning diagram illustrating the relative contributions of environmental predictors to explained variance in trait network metrics. AT, air temperature; Soil P, soil phosphorus; LD, litter depth.

Air temperature, air humidity, soil P, and litter depth collectively accounted for 84.2% of the variation in all plant trait network metrics (*P* = 0.011, **Figure 4A**). Individually, these factors accounted for 86.9% (litter depth), 63.3% (soil P), 50.9% (air temperature), and 32.9% (air humidity) of the variation **Figures 4B, C**), while their four-way joint effect contributed 0% **Figure 4B**). The joint effects of specific three-factor combinations were significant: air humidity, litter depth, and soil P contributed 61.4%; air temperature, litter depth, and soil P contributed 41.1%; air temperature, air humidity, and litter depth contributed 26.2%; and air temperature, air humidity, and soil P contributed 20.6% **Figures 4B, C**). In contrast, the joint effects of any two variables had no effect on the trait network metrics (0% explained variation) **Figures 4B, C**). Regarding correlations between specific metrics and environmental factors **Figure 4A**), average path length and graph diameter showed positive correlations with air temperature but negative correlations with litter depth and soil P. SRA and SRL were positively correlated with litter depth and soil P but negatively correlated with air temperature. Tree height was positively correlated with air temperature and negatively correlated with litter depth and soil P. SLA showed positive correlations with air temperature, air humidity, litter depth, and soil P. Conversely, modularity was negatively correlated with air temperature, air humidity, litter depth, and soil P **Figure 4A**).

### 3.4 Controls on species distribution

**Figure 5:**
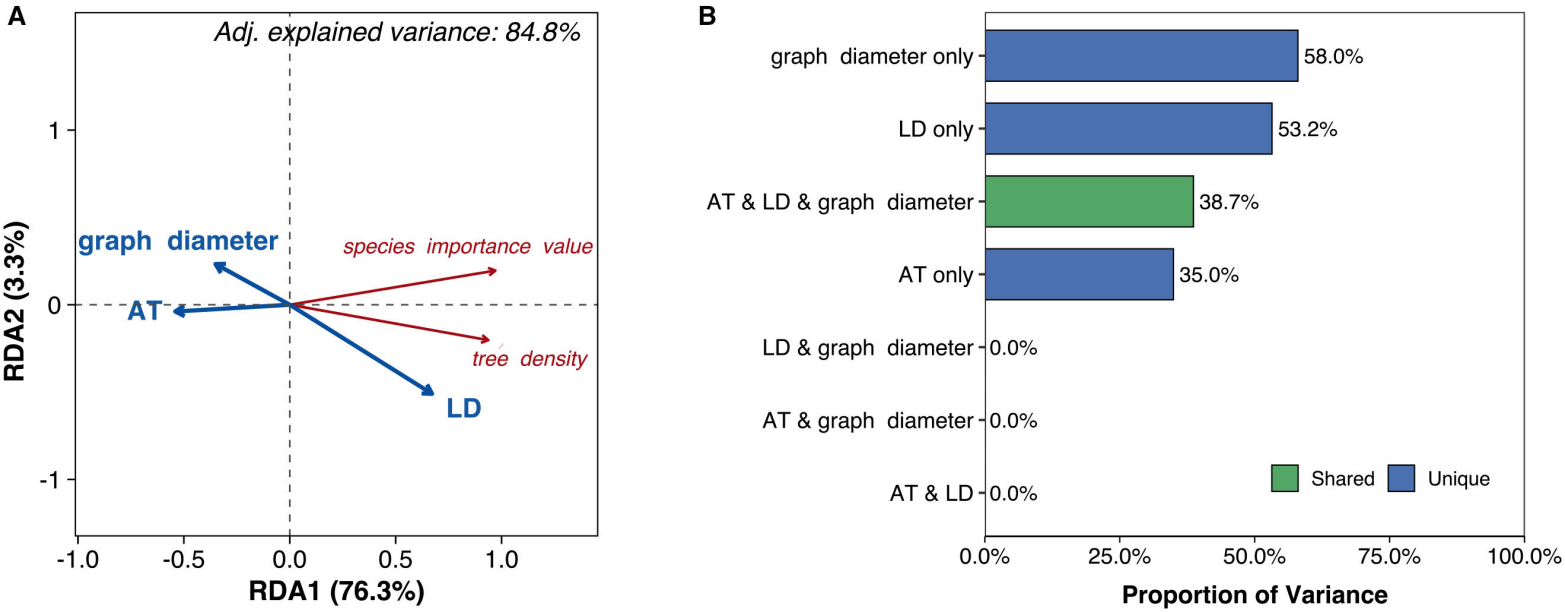
**(A)** Biplot from redundancy analysis (RDA) examining relationships between species distribution (red text labels) and environmental predictors (blue vectors). **(B)** Bar diagram illustrating the relative contributions of environmental predictors to explained variance in species distribution. AT, air temperature; LD, litter depth.

Air temperature, graph diameter, and litter depth collectively explained 84.8% of the variation in species distribution (*P* = 0.038, **Figure 5A**). Individually, these factors accounted for 35.0% (air temperature), 58.0% (graph diameter), and 53.2% (litter depth) of the variation, while their three-way joint effect contributed 38.7% **Figure 5B**). Regarding species-level responses **Figure 5A**), both species importance value and tree density exhibited negative correlations with air temperature and graph diameter, but positive correlations with litter depth (**Figure 5A**).

## 4 Discussion

Our study reveals that elevational gradients systematically reconfigure PTN architecture in woody saplings, primarily through litter-mediated microclimate buffering and soil phosphorus availability—mechanisms that outweigh direct climatic effects. These shifts in PTN structure subsequently mediate species distribution patterns. Below, we contextualize these findings in relation to our hypotheses and broader trait ecology.

### 4.1 Elevational restructuring of PTNs: Integration, modularity, and hub shifts

Supporting our first hypothesis, the significant decline in both average path length and graph diameter with increasing elevation **Figures 3B, C**) indicates that plant trait networks (PTNs) become more integrated under high-elevation stress. This aligns with theoretical predictions that cold environments select for tightly coordinated trait networks to optimize resource-use efficiency (Benavides et al., 2021; Segui et al., 2017). However, this contrasts with studies reporting reduced network connectivity under stressful conditions (Li et al., 2022; Rao et al., 2023). This inconsistency may stem from differences in study scale: previous findings often originated from broader (macroscopic) scales (He et al., 2020; Li et al., 2021), whereas our regional-scale study captured diverse microhabitats. Specifically, we observed negative relationships between litter depth/soil phosphorus (P) and both network diameter and average path length (**Figure 4A**), suggesting sapling PTNs become more integrated where litter is deeper, and soil P is higher at the tree bases. This finding contrasts with macroscopic-scale reports of declining soil nutrients with elevation (Weemstra et al., 2022). The deeper litter layer observed in colder areas (**Supplementary Figure S1h**) likely acts as an insulating layer for saplings, extending their suitable growth period. Enhanced litter accumulation further supports plant survival by reducing soil erosion, retaining moisture and nutrients, and providing critical habitat for microorganisms (Kaspari and Yanoviak, 2008). Furthermore, high-elevation saplings in our study, dominated by Rhododendron species, frequently form associations with ericoid mycorrhizal (ErM) fungi. These symbionts enhance survival in cold, acidic soils by enzymatically degrading organic matter, thereby increasing nutrient availability (Tedersoo et al., 2020). Critically, previous studies primarily focused on aboveground trait networks (Li et al., 2022; Matesanz et al., 2021). Given that plant growth requires integrated resources (light, carbon, water, nutrients; Weemstra et al., 2022), a whole-plant trait perspective is scientifically essential. Therefore, PTN integration appears strongly influenced by microenvironmental factors and plant mycorrhizal type, underscoring the need to incorporate these elements in future research.

Consistent with our second hypothesis, modularity peaked at mid-elevation **Figure 3A**). This inverted U-shaped pattern reflects intensified functional trade-offs at intermediate elevations, where plants must simultaneously balance traits for tolerance to cold/acidic stress with those for light/nutrient acquisition. Modularity decreased with increasing soil phosphorus, litter depth, air temperature, and air humidity **Figure 4A**). Crucially, litter depth showed little change or even a slight decrease from low to mid-elevations, while air humidity exhibited a U-shaped relationship, resulting in drier conditions at mid-elevations (**Supplementary Figure S1j**). Combined with the linear decrease in air temperature (**Supplementary Figure S1i**), these microenvironmental factors collectively drive the observed modularity peak at mid-elevation (**Figure 4A**), forcing trait decoupling into specialized modules to adapt to complex environmental demands (Li et al., 2021). At high-elevation extremes, higher humidity, soil P, and deeper litter layers reduce modularity (He et al., 2020). Conversely, at low elevations, higher temperatures and humidity also decrease modularity. Although litter depth and soil P are lower here, accelerated decomposition rates under warm, humid conditions enhance nutrient cycling (Dai et al., 2020), further reducing modularity. Therefore, plant trait network modularity is shaped by the interplay of multiple factors, with microenvironmental conditions playing a critical role. Future studies should incorporate additional soil nutrient metrics to test this framework.

The shift in hub traits from aboveground (height, SLA) to belowground (specific root length - SRL, specific root area - SRA) indicates changing resource limitations across the elevation gradient (**Figure 2**), supporting hypothesis H3. The increase in SRL and SRA with elevation (**Figures 3G, H**) signifies carbon reallocation towards efficient nutrient foraging—a critical adaptation to phosphorus-limited, acidic soils prevalent at higher elevations (Jian et al., 2022; Wang et al., 2023). This strategy is particularly evident in the dominant high-elevation Rhododendron species in our study. These plants form ericoid mycorrhizal (ErM) associations, which release specific enzymes to decompose litter and access nutrients (Tedersoo et al., 2020). High SRL/SRA allows efficient nutrient acquisition with low carbon investment, enabling rapid growth during the brief growing season at high elevations (Wang et al., 2025). Conversely, SLA remained a central hub trait across mid-elevations (1100–1700 m; **Figure 2**), reflecting persistent competition for light before nutrient constraints become the dominant limiting factor (Wang et al., 2022b). Therefore, the architecture of plant trait networks effectively reveals the core strategies plants employ to adapt to environmental challenges.

### 4.2 PTN efficiency drives species distribution

Species distribution exhibited a stronger correlation with network-wide efficiency – reflected by reduced graph diameter and path length (**Figure 5A**) – supporting hypothesis H4. This suggests that resource acquisition and utilization efficiency in saplings increases with elevation (Li et al., 2021), enhancing growth rates (Lei et al., 2025) and enabling high-elevation saplings to complete essential growth processes within their shorter favorable temperature window, thereby reducing mortality. This adaptation further explains the observed elevational increase in woody saplings with higher leaf carbon content (Wang et al., 2022b). Constructing highly connected trait networks requires substantial carbon investment (Rao et al., 2023), making saplings with greater leaf carbon reserves better equipped to establish within forest overstories.

Crucially, while hub traits within the network were environmentally sensitive (**Figure 4**), they did not directly correlate with sapling elevation distribution (**Figure 5**). This finding aligns with prior studies documenting weak trait-distribution relationships in species distribution modeling (Beissinger and Riddell, 2021; Lawlor et al., 2024). Three key factors likely explain this pattern: First, many studies assume linear trait-distribution relationships despite evidence of nonlinear associations (Beissinger and Riddell, 2021). Second, predictive capacity depends critically on trait selection—while leaf traits show no elevational correlation, plant size and hydraulic traits exhibit stronger distribution-predictive power (Maharjan et al., 2021; Lawlor et al., 2024; Estrada et al., 2016). Third, mycorrhizal mediation significantly influences outcomes; absorptive root traits predict elevational niches in ErM species but not in arbuscular or ectomycorrhizal species (Wang et al., 2025). This implies that shifts in individual traits or pairwise trait correlations alone are unlikely to alter species distributions or ecosystem functions (Kleyer et al., 2019). This network-level perspective clarifies why previous studies found limited predictive power in individual plant functional traits for community dynamics (Garcia Criado et al., 2023; van der Plas et al., 2020).

Instead, environmental pressures drive reorganization of the overall network structure, which subsequently impacts distribution and function (He et al., 2020). Supporting this framework, species distribution correlated negatively with air temperature (**Figure 5A**) – consistent with numerous studies – indicating that climate warming will likely drive an upward shift in regional woody plant distributions (Steinbauer et al., 2018; Lawlor et al., 2024). Future species-level research is needed to predict migration extent and direction for specific taxa. Furthermore, greater litter depth significantly increased species importance value and tree density (**Figure 5A**), identifying it as a previously overlooked facilitator of network efficiency. As established, the deep litter layer in cold, humid high-elevation environments acts as a critical thermal insulator for saplings, extending their viable growth period. Consequently, analyzing plant trait networks within their microenvironmental context emerges as a powerful approach for unraveling species distribution mechanisms under global change.

### 4.3 Theoretical reconciliation: Scale and organ integration matter

Our findings clarify contradictions in stress-PTN relationships: Whole-plant integration (leaves and roots) reveals increased PTN connectivity under elevation stress—contrasting studies focused solely on leaves (Matesanz et al., 2021). Litter’s microclimate role explains why high-elevation saplings sustain integrated networks despite cold stress: litter buffers temperature extremes and prolongs growth (Wang et al., 2022b). Soil P’s joint effects with litter (**Figure 4C**) underscore that nutrient availability enables resource-acquisitive root traits (SRL/SRA), facilitating network integration. Thus, PTNs capture context-dependent strategies where belowground organs and habitat modifiers (litter) dictate high-elevation adaptation.

### 4.4 Limitations and future directions

While elevation provided a natural environmental stress gradient, future research should test PTN responses in low-competition systems such as arid zones where modularity may dominate; experimentally manipulate key drivers (litter depth, soil phosphorus, and temperature) to isolate causal effects on PTN architecture; and track ontogenetic shifts in PTN organization across life stages from saplings to mature trees.

## 5 Conclusions

Elevation-driven stresses reconfigure sapling PTNs toward greater integration (shorter path lengths, smaller diameters), primarily mediated by litter depth (thermal buffering) and soil phosphorus (nutrient provisioning)—not direct climate effects. This integration optimizes whole-plant efficiency, explaining why species distribution correlates strongly with streamlined network architecture. While hub traits shift from leaves (light capture) to roots (nutrient foraging; SRL/SRA) with elevation, species distribution arises from system-wide optimization, not isolated hubs. Consequently, predicting community responses to global change requires, prioritizing litter-soil-climate interactions in PTN models, tracking whole-plant trait coordination across life stages, and recognizing microhabitat modifiers (litter) as critical resilience buffers.

## Data Availability

The original contributions presented in the study are included in the article/**Supplementary Material**. Further inquiries can be directed to the corresponding author.

## Supplementary material

Supplementary Table 1 Species names across different elevational gradients in our study.

Supplementary Figure 1 Variation in soil properties **(a–e, h)**, climate **(i–k)**, species importance value **(g)**, and tree density (l) with elevation. SN, soil nitrogen; SP, soil phosphorus; SD, soil depth; SpH, soil pH; SH, soil humidity; ST, soil temperature; LD, litter depth; AT, air temperature; AP, air pressure; AH, air humidity.

Supplementary Figure 2 Pairwise Pearson correlations among environmental factors. SN, soil nitrogen; SP, soil phosphorus; AH, air humidity; AT, air temperature; AP, air pressure; SD, soil depth; LD, litter depth; SH, soil moisture; ST, soil temperature; SpH, soil pH.

Supplementary Figure 3 Locations of sampling sites on Mt. Fanjingshan. T1 to T6 represent elevational gradients. At each gradient, one site is established, where a 150-meter transect line parallel to the contour lines is set. Along each transect, 10 sampling points are placed at 15-meter equidistant intervals.
