# Supplementary material for: Whole-plant trait networks reveal elevational optimization of resource strategies: integration drives distribution in woody saplings
Source: Front Plant Sci. 2025 Sep 11;16:1463237. doi: 10.3389/fpls.2025.1463237 (PMC12461259; doi:10.3389/fpls.2025.1463237)
Supplement: Supplementary Table 1 — Species names across different elevational gradients in our study. [file Supplementaryfile1.docx]

**Supplementary Materials for**

**Whole-plant trait networks reveal elevational optimization of resource strategies: Integration drives distribution in woody saplings**

**Table S1** Species names across different elevational gradients in our study.

| Elevation/m | Family | Genus | Species |
| --- | --- | --- | --- |
| 600 | Cornaceae | dendrobenthamia | *Dendrobenthamia_japonica* |
| 600 | Theaceae | Eurya | *Eurya_japonica* |
| 600 | Lauraceae | Litsea | *Litsea_elongata* |
| 600 | Lauraceae | Litsea | *Litsea_pedunculata* |
| 600 | Fagaceae | Quercus | *Quercus_engleriana_Seemen* |
| 1100 | Theaceae | Camellia | *Camellia_japonica* |
| 1100 | Fagaceae | Cyclobalanopsis | *Cyclobalanopsis_multinervis* |
| 1100 | Fagaceae | Cyclobalanopsis | *Cyclobalanopsis_sessilifolia* |
| 1100 | Ebenaceae | Diospyros | *Diospyros_kaki_var_silvestris* |
| 1100 | Lauraceae | Litsea | *Litsea_subcoriacea* |
| 1100 | Magnoliaceae | Michelia | *Magnolia_maudiae* |
| 1100 | Fagaceae | Quercus | *Quercus_engleriana_Seemen* |
| 1100 | Ericaceae | Rhododendron | *Rhododendron_seniavinii* |
| 1100 | Symplocaceae | Symplocos | *Symplocos_paniculata* |
| 1100 | Symplocaceae | Symplocos | *Symplocos_sumuntia* |
| 1100 | Ericaceae | Vaccinium | *Vaccinium_bracteatum* |
| 1100 | Caprifoliaceae | Viburnum | *Viburnum_cylindricum* |
| 1480 | Theaceae | Camellia | *Camellia_cuspidata* |
| 1480 | Fagaceae | Cyclobalanopsis | *Cyclobalanopsis_argyrotricha* |
| 1480 | Daphniphyllaceae | Daphniphyllum | *Daphniphyllum_oldhami* |
| 1480 | Lauraceae | Lindera | *Lindera_communis* |
| 1480 | Lauraceae | Lindera | *Lindera_fragrans* |
| 1480 | Lauraceae | Litsea | *Litsea_elongata* |
| 1480 | Lauraceae | Machilus | *Machilus_microcarpa* |
| 1480 | Ericaceae | Rhododendron | *Rhododendron_simsii* |
| 1480 | Rosaceae | Sorbus | *Sorbus_hemsleyi* |
| 1480 | Symplocaceae | Symplocos | *Symplocos_phyllocalyx* |
| 1480 | Ericaceae | Vaccinium | *Vaccinium_bracteatum* |
| 1700 | Theaceae | Camellia | *Camellia_cuspidata* |
| 1700 | Rosaceae | Cerasus | *Cerasus_tomentosa* |
| 1700 | Fagaceae | Cyclobalanopsis | *Cyclobalanopsis_argyrotricha* |
| 1700 | Theaceae | Eurya | *Eurya_japonica* |
| 1700 | Aquifoliaceae | Ilex | *Ilex_chinensis_Sims* |
| 1700 | Lauraceae | Laurus | *Laurus_nobilis* |
| 1700 | Symplocaceae | Symplocos | *Symplocos_sumuntia* |
| 1700 | Ericaceae | Vaccinium | *Vaccinium_bracteatum* |
| 1700 | Caprifoliaceae | Viburnum | *Viburnum_dilatatum* |
| 2000 | Cornaceae | Bothrocaryum | *Bothrocaryum_controversum* |
| 2000 | Theaceae | Camellia | *Camellia_cuspidata* |
| 2000 | Theaceae | Camellia | *Camellia_japonica* |
| 2000 | Aquifoliaceae | Ilex | *Ilex_chinensis_Sims* |
| 2000 | Magnoliaceae | Illicium | *Illicium_verum* |
| 2000 | Lauraceae | Litsea | *Litsea_elongata* |
| 2200 | Ericaceae | Enkianthus | *Enkianthus_quinque* |
| 2200 | Oleaceae | Ligustrum | *Ligustrum_lucidum* |
| 2200 | Lauraceae | Litsea | *Litsea_greeaniana* |
| 2200 | Rosaceae | Malus | *Malus_spectabilis* |
| 2200 | Ericaceae | Rhododendron | *Rhododendron_auriculatum* |
| 2200 | Ericaceae | Rhododendron | *Rhododendron_longicalyx* |

**Figure S1** Variation in soil properties (a-e, h), climate (i-k), species importance value (g), and tree density (l) with elevation. SN, soil nitrogen; SP, soil phosphorus; SD, soil depth; SpH, soil pH; SH, soil humidity; ST, soil temperature; LD, litter depth; AT, air temperature; AP, air pressure; AH, air humidity.

**Figure S2** Pairwise Pearson correlations among environmental factors. SN, soil nitrogen; SP, soil phosphorus; AH, air humidity; AT, air temperature; AP, air pressure; SD, soil depth; LD, litter depth; SH, soil moisture; ST, soil temperature; SpH, soil pH.

**Figure S3** Locations of sampling sites on Mt. Fanjingshan. T1 to T6 represent elevational gradients. At each gradient, one site is established, where a 150-meter transect line parallel to the contour lines is set. Along each transect, 10 sampling points are placed at 15-meter equidistant intervals.
